# Taiwan's Potential to Assist Developing Countries to Combat Infectious Diseases

**DOI:** 10.1371/journal.pmed.0030322

**Published:** 2006-07-25

**Authors:** Govindasamy Agoramoorthy, Minna J Hsu

**Affiliations:** **1**Tajen UniversityPingtungTaiwan; **2**National Sun Yat-sen UniversityKaohsiungTaiwan

The article on influenza in tropical regions by Viboud and colleagues outlines an alarming global burden of influenza, with an estimated one million annual deaths worldwide [ 1]. We would like to give an example of the immense potential of Taiwan to assist developing world communities in fighting against emerging infectious diseases in the near future.

Taiwan (area 36,000 km ^2^; population 23 million), officially known as the Republic of China, is located on the Tropic of Cancer. Taiwan lost its United Nation membership in 1971 to its rival, the People's Republic of China, but maintains official diplomatic relations with 25 countries and de facto relations with many nations. Taiwan has one of the highest levels of life expectancy in Asia, and it has eradicated infectious diseases such as the bubonic plague in 1948, smallpox in 1955, rabies in 1959, malaria in 1965, and polio in 2000. Taiwan became the first country in the world to implement a hepatitis B immunization program. Taiwan also initiated active vaccination programs against diptheria, pertussis, and tetanus in 1955, Japanese encephalitis in 1969, measles in 1978, hepatitis B in 1984, rubella in 1986, hepatitis A in 1995, and influenza in 1998. Valuable lessons may be learned from Taiwan's experience in public health and its response to disease outbreaks and crises.

Taiwan provides a modern, world-class health-care system to its people [ 2]. According to Taiwan Department of Health's statistics, the average life expectancy in 1951 was 53.38 years, and it increased to 73.35 years for men and 79.05 years for women in 2003. The Centre for Disease Control in Taiwan was established in 1999 to consolidate disease control resources. When an epidemic of enterovirus took the lives of 78 people in 1998, Taiwan responded by setting up disease surveillance [ 3].

To fight influenza, Taiwan embarked on a free influenza immunization program aiming to increase the coverage rate to 80% for those above the age of 65. There has been an increase in immunization of the elderly from 59.9% in 2002 to 68.4% in 2003, with medical care providers and disease control staff immunization up to 91.3%. Taiwan is committed to retaining vaccination production capability in the event of an emergency. The government is supporting a plan for the domestic production of influenza vaccines over the next few years as a part of Taiwan's readiness for an avian flu epidemic.

Taiwan is committed to work with the global medical community by contributing its resources and expertise. Unfortunately, it is not a member of the World Health Organization (WHO). It has therefore been excluded from the Global Outbreak Alert and Response Network (GOARN). During the time of the enterovirus outbreak, Taiwan did not get any assistance from the WHO but managed to combat the outbreak. The avian flu scare of 1997 was controlled in Hong Kong because of timely action, aided by the WHO. If Taiwan is to respond effectively to similar outbreaks of global epidemics in the future, it will certainly need the cooperation of the WHO. Although the WHO is a non-political agency, because of pressure from the People's Republic of China, which considers Taiwan as a renegade province, Taiwan has been marginalized. Regardless of this, Taiwan has contributed over US$180 million since 1995 in medical and humanitarian aid to 95 countries, and it plans to make further donations. Therefore, excluding Taiwan from the WHO's GOARN is counterproductive from both medical and ethical standpoints.

Taiwan, through its own efforts, has managed to improve public health remarkably, despite having no assistance from the WHO. It certainly has the potential to assist developing nations in the fight against emerging disease outbreaks, so other countries can seek Taiwan's expertise to fight against infectious diseases in the near future.
